# Position-Wise Gated Res2Net-Based Convolutional Network with Selective Fusing for Sentiment Analysis

**DOI:** 10.3390/e25050740

**Published:** 2023-04-30

**Authors:** Jinfeng Zhou, Xiaoqin Zeng, Yang Zou, Haoran Zhu

**Affiliations:** College of Computer and Information, Hohai University, Nanjing 210098, China; zhoujinfeng@hhu.edu.cn (J.Z.);

**Keywords:** sentiment analysis, deep neural networks, convolutional neural network, ResNet, Res2Net

## Abstract

Sentiment analysis (SA) is an important task in natural language processing in which convolutional neural networks (CNNs) have been successfully applied. However, most existing CNNs can only extract predefined, fixed-scale sentiment features and cannot synthesize flexible, multi-scale sentiment features. Moreover, these models’ convolutional and pooling layers gradually lose local detailed information. In this study, a new CNN model based on residual network technology and attention mechanisms is proposed. This model exploits more abundant multi-scale sentiment features and addresses the loss of locally detailed information to enhance the accuracy of sentiment classification. It is primarily composed of a position-wise gated Res2Net (PG-Res2Net) module and a selective fusing module. The PG-Res2Net module can adaptively learn multi-scale sentiment features over a large range using multi-way convolution, residual-like connections, and position-wise gates. The selective fusing module is developed to fully reuse and selectively fuse these features for prediction. The proposed model was evaluated using five baseline datasets. The experimental results demonstrate that the proposed model surpassed the other models in performance. In the best case, the model outperforms the other models by up to 1.2%. Ablation studies and visualizations further revealed the model’s ability to extract and fuse multi-scale sentiment features.

## 1. Introduction

Sentiment analysis (SA) is one of the most fundamental tasks in the field of natural language processing (NLP). With the support of massive subjective-opinion data and the development of artificial neural networks (ANNs), various neural networks, including recurrent neural networks (RNNs), memory networks, and convolutional neural networks (CNNs), have been widely applied in this field. In particular, following the remarkable success of CNNs across numerous fields, including computer vision, speech recognition, and signal processing, they have also been successfully applied to NLP tasks [1,2,3,4].

**Figure 1:**
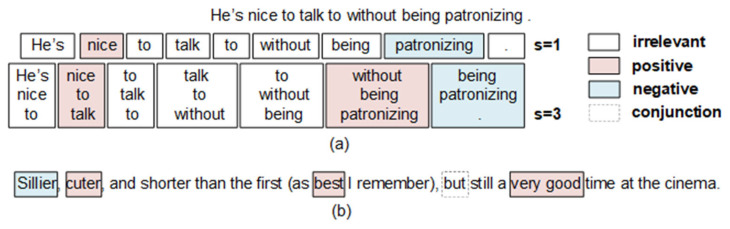
Impacts of multi-scale words and phrases on analyzing the sentiment of a text. (**a**) Limitations of the use of fixed scales to extract sentiment features. (**b**) Importance of jointly determining text sentiment by local sentiment words and phrases of different positions and scales.

One of the significant advantages of CNNs in SA is that they naturally learn coarse-to-fine multi-scale sentiment features using a stack of convolutional layers. Similarly, the text structure is hierarchical, and sentiment occurs in natural language in a multi-scale form. Most CNN-based models employ convolution filters with fixed window sizes to extract fixed-scale sentiment features [5,6,7]. However, the formation of a scale of sentiment features requires the flexible synthesis of various small-scale sentiment features. For example, the sentiment feature synthesis of “*nice to talk to without being patronizing*” (as shown in Figure 1a) preferably requires the sentiment features of “*nice*” (1-scale) and “*without being patronizing*” (3-scale). If the input feature scale is 1, the positive sentiment feature of “*nice*” and the negative sentiment feature of “*patronizing*” will be used as part of the input, which may introduce noise to the new sentiment feature. If the input feature scale is 3, the sentiment features of “*He’s nice to*” and “*nice to talk*” will be used as part of the input, which may add a large amount of unnecessary information to “*nice*” and weaken the response of the new sentiment feature to “*nice*”. Therefore, the interactions and fusion of multi-scale sentiment features are very important for learning large-scale sentiment features.

Furthermore, the sentiment of a text is jointly determined by local sentiment words or phrases of different positions and scales; conjunctions also play an important role. Most traditional CNNs obtain a global text sentiment representation by stacking convolutional and pooling layers [8,9,10]. This requires the resolution of two problems: fully using different scales of sentiment features to generate the text sentiment representation and reducing the loss of local detailed information in the convolution and pooling processes. Taking the sentence—“*Sillier, cuter, and shorter than the first (as best I remember), but still a very good time at the cinema*.”—as an example (as shown in Figure 1b), it has a positive global sentiment polarity and contains conjunctions, words, and phrases of different sentiment polarities. The source of its text sentiment should preferably include these features of 1-scale and 2-scale that highlight the sentiment of “*sillier*”, “*cuter*”, “*best*”, and “*very good*”, as well as the features that emphasize the semantics of “*but*”. For CNNs, if a text sentiment representation depends solely on the downstream layers, some information contained in small-scale sentiment features may be lost. Therefore, it is helpful for a task-friendly text sentiment representation to selectively reuse all scales of sentiment features.

Currently, there are two approaches to alleviate the above limitations: convolution filters with various window sizes in a layer and densely connected layers [6,11,12]. The first approach utilizes filters with different window sizes to extract multi-scale sentiment features. However, it is difficult to find the optimized combination of different window sizes. The interactions of sentiment features from different window sizes have also not been fully exploited, resulting in an insufficient ability to learn multi-scale sentiment features. A large-scale sentiment feature can be elegantly constructed through interactions between various small-scale features without relearning redundant features. The second approach can form a large-scale sentiment feature using various small-scale sentiment features and gracefully reuse all scales of sentiment features using dense connections [13,14]. However, this approach requires the stacking of multiple layers or blocks to obtain multi-scale sentiment features over a large range, resulting in a sharp increase in memory because of the dense connections. Recently, [15] proposed Res2Net in computer vision. Residual-like connections may provide interactions among various small-scale sentiment features to help synthesize large-scale sentiment features. However, these connections are implemented by direct addition, which cannot optimally select the appropriate sentiment features.

In this study, a new CNN-based model is proposed to adaptively learn more scales of sentiment features and fuse them selectively into a task-friendly text sentiment representation. Specifically, it comprises two important modules: a position-wise gated Res2Net (PG-Res2Net) module and a selective fusing module. First, each text is fed into the PG-Res2Net module to obtain different scales of sentiment features over a large range. Each block in the module uses multi-way convolution, residual-like connections, and position-wise gates to implicitly learn multi-scale sentiment features within a certain range. Multi-way convolution enables the module to stack a few residual blocks to obtain multi-scale sentiment features over a wide range. Residual-like connections also provide a bridge for the interactions between multi-scale sentiment features. Position-wise gates optimize the interactions. Furthermore, the selective fusing module integrates these sentiment features to generate a task-friendly text sentiment representation. Specifically, its dense-like connections reuse these features, and its selection operation selects the appropriate information from these features to generate a text sentiment representation. Finally, the text sentiment representation is fed into a classifier for prediction. These two modules enable the model to achieve competitive results on multiple SA datasets, particularly document-level datasets.

The major contributions of this work are as follows:

(1) This paper proposed a PG-Res2Net module to learn different scales of sentiment features over a large range. In contrast to convolution filters with fixed window sizes or dense connections for learning sentiment features, a single residual block in the module can learn multi-scale sentiment features within a certain range. Essentially, the module achieves the first selection of multi-scale features based on local statistics.
(2) Moreover, a selective fusing module is proposed to fully reuse and selectively fuse all scales of sentiment features. This is the second selection of multi-scale sentiment features based on global statistics. The module also effectively alleviates the loss of local detailed information caused by the convolution operation.
(3) The model is extensively evaluated on five datasets. The experimental results demonstrated the competitive performance of the model on these datasets. In the best case, the model outperforms the other models by up to 1.2%. In addition, visualizations and ablation studies demonstrated the effectiveness of the model.

The rest of this paper is organized as follows. Section 2 presents a brief survey of related work. A detailed description of the proposed model and the knowledge relevant to the model are presented in Section 3. Section 4 presents experimental results, ablation studies, and visual analysis. Finally, Section 5 is the conclusion that summarizes the work of this paper.

## 2. Related Work

SA is typically represented as a tuple (*target*, *sentiment*, *opinion holder*, and *time*). The element *target* is represented as a tuple (*category*, *entity*, and *aspect*), and the element *sentiment* is represented as a tuple (*type*, *intensity*, and *opinion terms*). Currently, most SA methods focus on these tuples or part of their elements. For example, structured sentiment analysis attempts to predict structured sentiment graphs by discovering all opinions and focusing on the whole entire tuple of SA [16]. As another example, emotion cause analysis is the detection of potential causes for certain emotional expressions in a text [17]. This is a study of the tuple *sentiment*. In addition, many interactive correlations between different elements can be shared by incorporating subtasks for handling combinations of different elements. As examples, Fei [18] and Yan et al. [19] proposed unified frameworks for aspect-based SA tasks. Our study focuses on the elements *intensity* and *opinion terms* within the tuple *sentiment* using sentiment modeling. This section presents some multi-scale sentiment modeling methods, including CNNs, residual networks (ResNets), and attention mechanisms relevant to this study.

### 2.1. CNNs and ResNets in SA Tasks

CNNs are suitable for extracting text sentiment because they naturally correspond to the multi-scale form of sentiment occurrence and the hierarchical structure of texts. Generally, a filter with a fixed window size learns fixed-scale sentiment features. Kim [11] first used multiple filters with different window sizes in a single convolutional layer to learn the sentiment features at several fixed scales. Subsequently, CNNs developed more varieties in SA. The effectiveness of the convolutional filters is an important factor in ensuring the quality of the extracted features. To enhance the ability to extract important semantic features, Yao and Cai [20] used the naïve Bayes algorithm to initialize convolutional filters to identify the positions of important semantic information before training. The concept of multi-scale was also developed. A new feature extraction method was proposed by Soni et al. [21]. The method constructed a text as a three-dimensional paragraph matrix and explicitly applied two-dimensional convolution operation to the matrix to obtain intra-sentence and inter-sentence multi-scale features. Dependency trees model the syntactic relationship between words and are used to improve the performance of models for SA. Graph convolutional networks (GCNs), which are an adaptation of the CNNs for handling unstructured data, can facilitate the handling of dependency trees. Zhang et al. [22] built a universal-syntax GCN over the syntactic dependencies with labels to achieve the goal of navigating richer syntax information for the best aspect-based SA robustness. With the development of deep learning, several strategies and approaches have been proposed for improving the ability of CNNs to extract sentiment features [23]. Of these, residual learning is an important approach and has been applied to SA tasks to improve the ability of CNNs to extract sentiment features. Conneau et al. [24] proposed VD-CNN, which is a pure ResNet that uses up to 29 layers to extract more and larger-scale sentiment features with minimal computational cost. Without relying solely on stacking convolutional layers, a CNN with dense connections was proposed by Wang et al. [6] to reuse existing multi-scale sentiment features and flexibly generate larger-scale features. Yan et al. [12] used a feature extraction block based on a convolution operation and a feature extraction block with dense connections as its feature extraction module, and their parallelism saved training time and reduced training iterations.

However, the aforementioned models must predefine and optimize the window sizes of convolutional filters, lack the interaction between sentiment features, or rely on deeper networks to synthesize more and larger-scale sentiment features. In addition, most of these models gradually lose more local information owing to convolution or pooling operations.

### 2.2. Attention Mechanisms in SA Tasks

Attention mechanisms are to simulate human attention and make models focus on task-related information to reduce computational complexity and improve performance [25]. Many models have attached different attention mechanisms to solve a wide range of SA tasks. An important role of attention mechanisms is to discover keywords and phrases that strongly contribute to sentiment classification. Lee et al. [26] implemented a word attention mechanism based on weakly supervised learning to identify keywords. Attention mechanisms can also capture behaviors related to the syntactic and semantic structures of a text [27]. Vaswani et al. [28] completely abandoned RNN and CNN structures and used only a multi-head self-attention mechanism to learn global dependencies for generating a text representation that is more relevant to semantics. Ambartsoumian and Popowich [29] explored two methods for combining multi-head self-attention based on the analysis of the characteristics of self-attention mechanisms and achieved competitive accuracy in multiple SA tasks. Attention mechanisms have also been widely used to enhance the aspect–opinion binding, which essentially solves aspect-based SA tasks. In order to pay more attention to the opinion expressions of aspects, Tan et al. [30] constructed a multi-graph fusion network based on GCNs and multiple attention mechanisms to exploit the syntax dependency relation label information and the affective semantic information of words. In addition, gating mechanisms, which control the flow of information through gating units according to the needs of a specific task, are an implementation form of attention mechanisms. Xue and Li [9] applied Tanh-ReLU gating units to the multi-scale sentiment features extracted by the top layer of a CNN to accurately select aspect- or target-related sentiment information. Liu et al. [31] used a convolutional layer and a gating mechanism before a pooling layer for generating attention weights, which helped the pooling layer to find genuinely critical features. Ren et al. [2] developed a gating mechanism similar to long short-term memory (LSTM) networks to control the flow of information between convolutional layers and improve the ability to extract features. Choi et al. [32] used gate mechanisms for the automatic calculation of the importance degrees of sentences in documents.

## 3. Material and Methods

### 3.1. Task Modeling

The sentiment classification of texts can be formulated as follows: given an input text *S* = {Wrd_1_, Wrd_2_ … Wrd_L_} comprising *L* words, where each element denotes a word of a sentence, our task is to construct a sentiment classifier that predicts the whole sentiment polarity *y*∈***O*** of *S*, where $O=\left\{O_{1},O_{2},\cdots O_{C}\right\}$ denotes the sentiment categories of the current task.

### 3.2. Overview

This section presents the novel and effective model, which is fundamentally designed to obtain text sentiment representations from multi-scale sentiment features at a wide range. The creditable multi-scale sentiment features achieved through the interactive fusion of existing features provide the actual meaning of every token in optimized contexts. Then, high-quality text sentiment representations generated through selectively fusing all scales of sentiment features better retain sentiment information for improved sentiment prediction.

**Figure 2:**
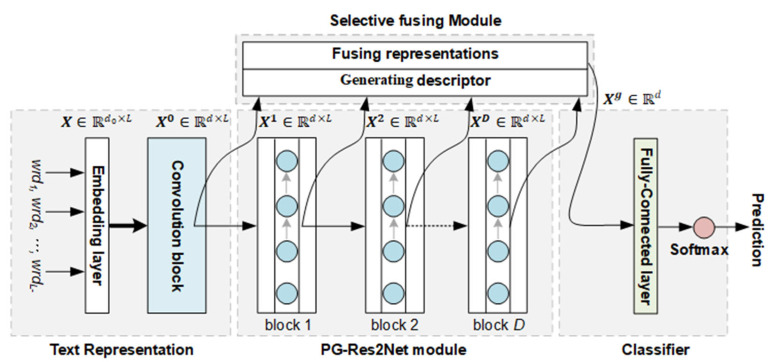
Overview framework of the proposed model.

As illustrated in Figure 2, the framework of the model is divided into four processing parts. First, an embedding layer and a convolution block are used to map the text into a text matrix. The text matrix is then fed into a position-wise gated Res2Net (PG-Res2Net) module to obtain different levels of sentiment representations, each of which comprises a certain range of multi-scale sentiment features. Subsequently, the text matrix and these sentiment representations are sent to a selective fusing module through dense-like connections. The selective fusing mechanism of the module is applied to selectively fuse all sentiment features in these representations into a text sentiment representation. Finally, the representation is sent into a classifier for prediction.

### 3.3. Text Representation

Given a text {Wrd_1_, Wrd_2_ … Wrd_L_} of length *L*, each word is first transformed into a word vector. Let $x_{i}\in {\mathbb{R}}^{d_{0}}$ denote the *d*_0_-dimensional pre-trained word vector of Wrd_i_, and the text is represented as an embedding matrix ***X*** by an embedding layer:

(1) $$\begin{array}{c} X={\left[\begin{array}{ccc} x_{1}, & \cdots ,x_{i},\cdots & x_{L} \end{array}\right]}_{d_{0}\times L}, \end{array}$$

Using pre-trained word vectors can improve the performance in the absence of a large supervised training set [33]. 

To facilitate the operation of the residual blocks in the subsequent PG-Res2Net module, a convolution block projects the feature dimension of ***X*** from *d*_0_ to *d* and outputs a text matrix $X^{0}\in {\mathbb{R}}^{d\times L}$, which is formulated as follows:

(2) $$X^{0}=ReLU\left(BN\left(conv\left(X,\;W^{0}\right)\right)\right)$$

where *conv*(•) is a 1D convolution operation, *BN*(•) is batch normalization [34], and *ReLU* is a rectified linear unit [35]. $W^{0}\in {\mathbb{R}}^{d\times d_{0}\times 1}$ is the learnable weight.

### 3.4. PG-Res2Net Module

**Figure 3:**
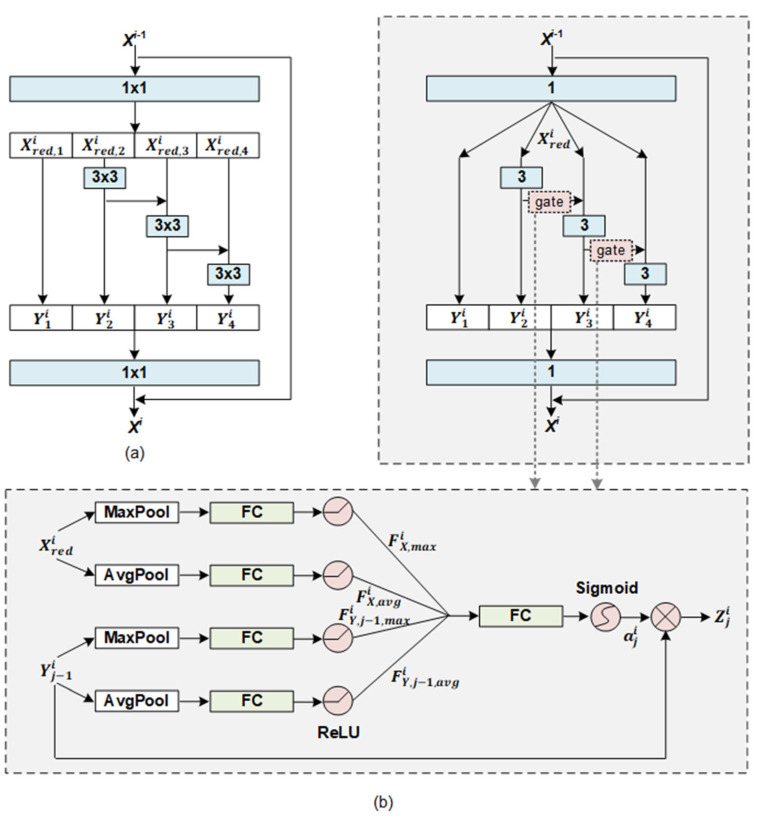
Structures of residual blocks in the two modules: (**a**) Res2Net and (**b**) PG-Res2Net.

Conventional CNN-based models lack the interaction between multi-scale sentiment features, and the scale range of the sentiment features relies solely on the network depth of these models. Following Res2Net and its variants in computer vision and other fields [15,36], a PG-Res2Net module was proposed for SA tasks. It has a strong ability to effectively and efficiently learn more and larger-scale sentiment features. For comparison, the structures of the residual blocks in the Res2Net and PG-Res2Net modules are illustrated in Figure 3a,b, respectively. Notably, the number of convolution ways *S* is set to 4, 1 × 1 denotes a 2D convolution with window size 1 × 1, and “1” denotes a 1D convolution with window size 1. “FC” is the abbreviation for “Fully-Connected Layer”. As shown in the two images, the most prominent difference between the two modules is that the residual-like connection between the two convolution ways in the Res2Net module is direct addition, whereas the residual-like connection in the PG-Res2Net module has a gate before addition. Different positions in the same text have different optimal scales to form new scale features. We expect that the gating mechanism gives priority to features at these optimal scales and suppresses less relevant features and then enhances the quality of new scale features.

As shown in the upper part of Figure 3b, for residual block *i*, its input ***X***^*i*−1^ is first compressed by a convolution sub-block to reduce the computational cost and avoid overfitting. The calculation of the sub-block is as follows:

(3) $$X_{\mathrm{red}}^{i}=ReLU\left(BN\left(conv\left(X^{i-1},\;W_{\mathrm{red}}^{i}\right)\right)\right),$$

where $X_{\mathrm{red}}^{i}\in {\mathbb{R}}^{\left(d/r\right)\times L}$ denotes the output of the sub-block, *r* is the dimension reduction ratio, and $W_{\mathrm{red}}^{i}$ is the learnable weight. Notably, $i\in \left[1,D\right]$, where *D* is the number of residual blocks in the PG-Res2Net module.

Subsequently, $X_{\mathrm{red}}^{i}$ is fed into *S* convolution ways, respectively. As shown in Figure 3a,b, unlike the original Res2Net in computer vision tasks, $X_{\mathrm{red}}^{i}$ is not grouped because text semantics requires a complete feature space. The output $Y_{j}^{i}\in {\mathbb{R}}^{\left(d/r\right)\times L}$ of way *j* is derived as follows:

(4) $$Y_{j}^{i}=\left\{\begin{array}{cc} X_{\mathrm{red}}^{i}, & \;j=0\\ ReLU(BN\left(conv(X_{\mathrm{red}}^{i},W_{j}^{i})\right)), & \;j=1\\ ReLU(BN\left(conv(X_{\mathrm{red}}^{i}+Z_{j-1}^{i},W_{j}^{i})\right)), & \;1<j\leq S \end{array}\right.$$

(5) $$Z_{j}^{i}=Y_{j}^{i}⊗a_{j}^{i},$$

where $Z_{j}^{i}\in {\mathbb{R}}^{\left(d/r\right)\times L}$ is the amount of $Y_{j}^{i}$ flowing into way *j* + 1 through the position-wise gate $a_{j}^{i}$, and ⊗ denotes position-wise multiplication. $W_{j}^{i}\in {\mathbb{R}}^{(d/r)\times \left(d/r\right)\times 3}$ is the learnable weight. To improve the flexibility of the residual block in synthesizing a large-scale feature from various small-scale features, $Z_{j}^{i}$ is implemented using a position-wise gate. Its operation is illustrated in the lower part of Figure 3b. The gate considers the statistics of both $X_{\mathrm{red}}^{i}$ and $Y_{j-1}^{i}$ as references and prioritizes each position of $Y_{j-1}^{i}$. These priorities can highlight the sentiment information relevant to the feature extraction of way *j* and suppress less relevant information. Particularly, the information from $X_{\mathrm{red}}^{i}$ and $Y_{j-1}^{i}$ is aggregated to generate four independent feature descriptors: $F_{Y,j-1,\mathrm{avg}}^{i}$, $F_{Y,j-1,\max}^{i}$, $F_{X,\mathrm{avg}}^{i}$, and $F_{X,\max}^{i}$. The calculation process is as follows:

(6) $$F_{Y,j-1,\mathrm{avg}}^{i}=ReLU\left(W_{Y,\mathrm{avg},j-1,}^{i}AvgPool\left(Y_{j-1}^{i}\right)+b_{Y,\mathrm{avg},j-1}^{i}\right)$$

(7) $$F_{Y,j-1,\max}^{i}=ReLU\left(W_{Y,\max,j-1}^{i}MaxPool\left(Y_{j-1}^{i}\right)+b_{Y,\max,j-1}^{i}\right)$$

where *AvgPool* and *MaxPool* are the average-pooling and max-pooling operations in the feature dimension, respectively. $W_{Y,\mathrm{avg},j-1}^{i}\in {\mathbb{R}}^{(L/\gamma )\times L}$, $W_{Y,\max,j-1}^{i}\in {\mathbb{R}}^{\left(L/\gamma \right)\times L}$, $b_{Y,\mathrm{avg},j-1}^{i}\in {\mathbb{R}}^{L/\gamma }$, and $b_{Y,\max,j-1}^{i}\in {\mathbb{R}}^{L/\gamma }$ are the learnable weights. γ is the reduction ratio for compressing the dimensions of these descriptors and for avoiding overfitting. $F_{X,\mathrm{avg}}^{i}$ and $F_{X,\max}^{i}$ are derived in a manner similar to $F_{Y,j-1,\mathrm{avg}}^{i}$ and $F_{Y,j-1,\max}^{i}$. Notably, all of these are processed separately because their functionalities are not symmetric. All descriptors are then concatenated to produce $a_{j}^{i}\in {\mathbb{R}}^{L}$ as follows:

(8) $$a_{j}^{i}=Sigmoid\left(W_{\mathrm{gate},j}^{i}\left[F_{Y,j-1,\mathrm{avg}}^{i},F_{Y,j-1,\max}^{i},F_{X,\mathrm{avg}}^{i},F_{X,\max}^{i}\right]+b_{\mathrm{gate},j}^{i}\right)$$

where $W_{\mathrm{gate},j}^{i}\in {\mathbb{R}}^{L\times \left(4*L/\gamma \right)}$ and $b_{\mathrm{gate},j}^{i}\in {\mathbb{R}}^{L}$ are the learnable weights. *Sigmoid* is an activation function.

Finally, to better fuse the multi-scale sentiment features extracted by *S* convolution ways into the sentiment representation ***X^i^*** of residual block *i* and to ensure that the input and output dimensions of the block are the same, these features are concatenated and fed into a convolution sub-block. $X^{i}$ is calculated as follows:

(9) $$X^{i}=ReLU\left(BN\left(conv\left(\left[\begin{array}{ccc} Y_{1}^{i}, & Y_{2}^{i},\cdots & Y_{S}^{i} \end{array}\right],W_{\mathrm{fuse}}^{i}\right)\right)+X^{i-1}\right)$$

where $W_{\mathrm{fuse}}^{i}\in {\mathbb{R}}^{d\times \left(d*S/r\right)\times 1}$ is the learnable weight.

There is a remarkable advantage of the PG-Res2Net module: Residual-like connections and position gates provide better interactions between existing multi-scale sentiment features to enhance the quality of new scale features. In fact, the first selection of multi-scale sentiment features is completed based on the guidance of local statistics. A new scale sentiment feature essentially stores the appropriate information contained in the different scales of the sentiment features.

### 3.5. Selective Fusing Module

A residual block in the PG-Res2Net module generates a level of sentiment representation containing multi-scale sentiment features within a limited range, and stacking multiple residual blocks enables the production of different levels of sentiment representations containing more multi-scale sentiment features over a large range. However, only the sequential connections between these blocks may not flexibly and accurately handle language composition. Drawing on the ideas of dense connections and selective kernel convolution [37,38], a selective fusing module was proposed. Its dense-like connections reuse all existing sentiment representations, and its selection operation adaptively adjusts the contribution of these sentiment representations to produce a text sentiment representation.

**Figure 4:**
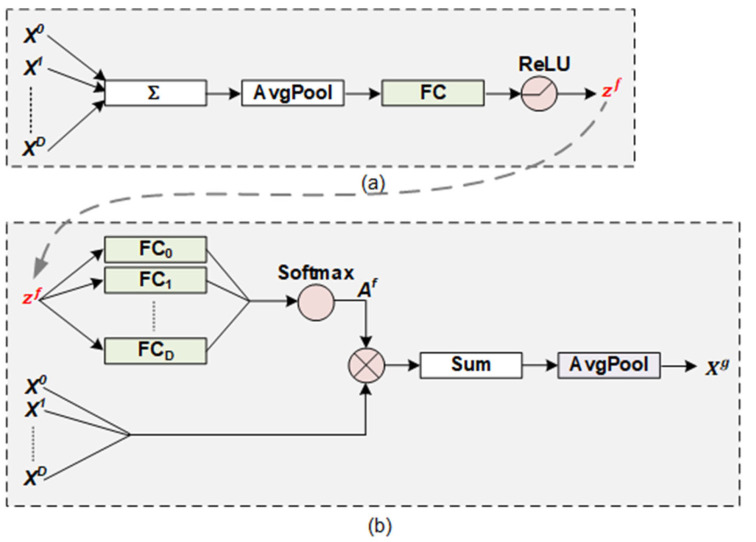
Structure of the selective fusing module. It includes two key operations: (**a**) generating a guidance descriptor and (**b**) fusing all sentiment representations based on the descriptor.

As shown in Figure 4a, the module first takes as input all levels of sentiment representations from the first convolution block and all residual blocks to generate a descriptor $z^{f}\in {\mathbb{R}}^{d}$. The descriptor provides global information as a guide for selection. Its calculation is formulated as follows:

(10) $$z^{f}=ReLU\left(BN(W_{1}^{f}AvgPool(\overset{D}{\underset{l=0}{\sum }}X^{l})+b_{1}^{f})\right)$$

where $W_{1}^{f}\in {\mathbb{R}}^{d\times d}$ and $b_{1}^{f}\in {\mathbb{R}}^{d}$ are the learnable weights. The module then uses soft selection, which is guided by *z^f^*, to select different sentiment information into a text representation $X^{g}\in {\mathbb{R}}^{d}$. This process is shown in Figure 4b. Particularly, $A^{f}\in {\mathbb{R}}^{\left(D+1\right)\times d}$ is a selective matrix, and any vector $a_{i}^{f}\in {\mathbb{R}}^{d}$ in the matrix represents the selective weights of *X^i^* in the feature dimension. The selective matrix is formulated as follows:

(11) $$A^{f}={\left[a_{0}^{f},a_{1}^{f},\cdots ,a_{d}^{f}\right]}_{\left(D+1\right)\times d}$$

(12) $$a_{i}^{f}=Softmax\left(W_{2,i}^{f}z^{f}\right)=\frac{exp\left(W_{2,i}^{f}z^{f}+b_{2,i}^{f}\right)}{\sum_{j=0}^{D}exp\left(W_{2,j}^{f}z^{f}+b_{2,j}^{f}\right)}$$

where *Softmax* is a normalized exponential function and *exp*(•) is an exponential function based on the natural constant *e*. $W_{2,i}^{f}\in {\mathbb{R}}^{d\times d}$ and $b_{1,i}^{f}\in {\mathbb{R}}^{d}$ are the learnable weights. Finally, ***X^g^*** is defined as follows:

(13) $$X^{g}=AvgPool(Sum\left(\left[X^{0},X^{1},\cdots X^{D}\right]⊗A^{f}\right))$$

where ⊗ is a level-wise product, and *Sum* is a sum function on the level dimension.

Each sentiment representation contains a certain range of selected multi-scale sentiment features. Essentially, the selective fusing module performs the second selection for all multi-scale sentiment features based on global statistics.

### 3.6. Objective Function

The classifier in our model was implemented using one fully-connected layer and used *X^g^* as its input. It outputs the prediction $y\in {\mathbb{R}}^{c}$ as follows:

(14) $$y=Softmax\left(W^{c}X^{g}+b^{c}\right)$$

where $W^{c}\in {\mathbb{R}}^{C\times d}$ and $b^{c}\in {\mathbb{R}}^{C}$ are the learnable weights. *C* is the number of sentiment categories in a dataset. The cross-entropy function *ε* is used as the training objective and minimized as follows:

(15) $$\varepsilon =-\overset{C}{\underset{i=1}{\sum }}\hat{y_{i}}*log\left(y_{i}\right)$$

where $\hat{y}\in {\mathbb{R}}^{C}$ denotes the referenced distribution.

In this model, the supervision signals are more directly propagated back to the upstream blocks through dense-like connections. Such connections force upstream blocks to learn task-friendly sentiment features, also known as “deep supervision” [37]. Given a sample, the gradient $\frac{\partial \varepsilon }{\partial X^{i}}$ is decomposed into *D* – *i* + 1 additive terms as follows:

(16) $$\frac{\partial \varepsilon }{\partial X^{i}}=\frac{\partial \varepsilon }{\partial y}\frac{\partial y}{\partial X^{g}}\frac{\partial X^{g}}{\partial X^{i}}+\frac{\partial \varepsilon }{\partial y}\frac{\partial y}{\partial X^{g}}\frac{\partial X^{g}}{\partial X^{i+1}}\frac{\partial X^{i+1}}{\partial X^{i}}+\cdots +\frac{\partial \varepsilon }{\partial y}\frac{\partial y}{\partial X^{g}}\frac{\partial X^{g}}{\partial X^{D}}\frac{\partial X^{D}}{\partial X^{i}}$$

(17) $$=\frac{\partial \varepsilon }{\partial y}\frac{\partial y}{\partial X^{g}}\left(\overset{D}{\underset{m=i}{\sum }}\frac{\partial X^{g}}{\partial X^{m}}\overset{m-1}{\underset{n=i}{\prod }}\frac{\partial X^{n+1}}{\partial X^{n}}\right)$$

where $i\in \left[0,\;D\right]$. The first term of Equation (16) indicates that the supervision information is directly propagated back to any upstream block *i* through only a few blocks or layers. Therefore, the block is forced to learn directly under the supervision signals. These additive terms also intuitively show that the training behavior is similar to the simultaneous training of a series of neural networks, the structures of which range from shallow to deep. In this manner, the learning of sentiment features is carried out under multiple supervision signals from multiple neural networks. These features better consider both feature synthesis and direct task purpose, which are reflected in the two terms inside and outside the brackets in Equation (17).

## 4. Results and Discussion

This section first describes the five public datasets used in our experiments, as well as the experimental setup and models for comparison. Next, the experimental results of the proposed model and other models on these datasets are presented. Finally, the effectiveness of the model is demonstrated through ablation studies and visualization.

### 4.1. Datasets

To verify the performance of the model in short-text-level and document-level SA tasks, the experiments were conducted on five datasets. The binary-category short-text-level datasets included MR [39] and SST-2 [40], and the multi-category document-level datasets consisted of Yelp.F [7], Sports & Outdoors (S&O), and Toys & Games (T&G) from SNAP [41].

- MR: The dataset was built by searching for movie reviews from review websites. In this dataset, 10,662 samples are separated into two categories.
- SST-2: The dataset is a binary version of the Stanford Sentiment Treebank dataset, which is an extension of MR. It comprises 9163 samples, which are separated into two categories.
- Yelp.F: The Yelp review dataset was obtained from the 2015 Yelp Dataset Challenge. It has five-star polarity labels. Each star label contains 130,000 training samples and 10,000 testing samples.
- S&O and T&G: These two datasets contain product reviews and metadata from SNAP, including 142.8 million reviews from Amazon. In this study, only reviews of Sports & Outdoor and Toy & Game products were used.

The complete details and statistics of these datasets are listed in Table 1. Note that S&O and T&G have no standard training/test split, and their split refers to [42].

### 4.2. Models for Comparison

To evaluate the performance of the model, it was compared with baseline and state-of-the-art models. The baseline methods are as follows:

- *Bi-LSTM* [43] directly inputs the entire document as a single sequence into a bi-directional LSTM network for SA.
- *HAN* [44] uses hierarchical attention networks to classify documents.
- *Classical CNN* [11] uses multiple filters with different window sizes in a single convolutional layer to learn the sentiment features.
- *VDCNN* [24] uses only small convolution and pooling operations at the character level with a depth of 29 convolutional layers.
- *Word-DenseNet* [45] is an adaptation of DenseNet for text classification.

The state-of-the-art models are as follows:

- *HUSN* [46] utilizes user review habits to enhance an LSTM-based hierarchical neural network for SA.
- *CAHAN* [47] is a modification of HAN that can make context-aware attentional decisions.
- *AGCNN* [31] introduces an attention-gated layer before the pooling layer to help the CNN focus on critical abstract features.
- *TextConvoNet* [21] applies multidimensional convolution to extract inter-token and inter-sentence N-gram features.
- *DenseNet with multi-scale feature attention* [6] is an improved version of DenseNet and is equipped with multi-scale feature attention.
- *SAHSSC* [48] is a self-attentive hierarchical model for text summarization and sentiment classification.
- *Sentiment-Aware Transformer* [49] is a new type of transformer model designed to predict both word and sentence sentiment.

### 4.3. Experimental Setup

The experimental setup of the proposed model involved three parts:

(1) **Input.** Data preprocessing was performed because the datasets were obtained from web reviews and had complex and arbitrary characteristics. Anomalous symbols were eliminated, and upper-case letters were converted to lower-case letters. A word embedding corpus pre-trained by GloVe was used [50]. Words in a target dataset that were not in the corpus were initialized using a random vector with element values between −0.01 and 0.01. Because the input of the model requires a constant length *L*, all samples whose length was not *L* were padded with zero vectors or truncated. In the experiments, *L* was set to 50 for MR and SST-2 and 500 for the other datasets.
(2) **Architecture configuration.** The feature dimension *d* of the output of the first convolutional block was set to 128. For the PG-Res2Net module, the reduction ratio γ was set to 2, and the number *S* of convolution ways of a residual block was set to 4. The number *D* of residual blocks was set to 2 for MR and SST-2, 4 for S&O and T&G, and 7 for Yelp.F. *S* and *D* were determined by the experimental results.
(3) **Training setting.** The objective function was minimized by stochastic gradient descent (SGD) with a batch size of 256, a learning rate of 0.01, and a momentum of 0.99. For all datasets except SST-2, the learning rate dropped to 0.1 times every 5 epochs. For SST-2, the period was 10 epochs. *L*_2_ regularization was also added to the objective function, and its coefficient was set to 0.0001. Random dropout [51] with a drop rate of 0.5 was applied to the input of the classifier. The training processes lasted for at most 20 epochs on all datasets, and all experiments were conducted using PyTorch v1.9 (Linux Foundation, San Francisco, CA, USA).

In the experiments, the above datasets were not processed by any pre-trained transformer model, such as BERT [52]. There are two reasons. First, the above datasets contain numerous long texts. The memory usage and computational complexity caused by the self-attention mechanism in pre-training models grow quadratically with the text length [53]. This can lead to excessive costs when processing long texts. Second, the proposed model aims to improve the ability to extract credible features, while pre-training models are usually used to initialize the feature vector for each word in the SA tasks. Therefore, whether or not pre-training models are used does not affect the demonstration for the innovation of the proposed model. In essence, the modules in the proposed model can be easily incorporated into several existing CNN-based models to improve their ability to extract multi-scale features. 

### 4.4. Experimental Results

The results of the proposed model and the other models for the five datasets are listed in Table 2. The proposed model achieved superior or comparable results to all other models. For the Yelp.F, S&O, and T&G datasets, the proposed model achieved the best accuracy, which was at least 0.5%, 1.2%, and 0.7% higher than those of the other models, respectively. Most of the samples in the three datasets are at the document level and have more complex sentiment semantic dependencies than short texts. Compared with those RNNs, the proposed model exhibited the ability to explicitly capture more and larger-scale sentiment features. Compared with those shallow CNNs, the proposed model could flexibly synthesize sentiment features on various scales and alleviate the problem of sentiment information utilization. Compared with other ResNets, the proposed model improved the interactions between multi-scale features and exhibited the capability to fuse different scales of sentiment features. For those transformer-based models, their self-attention may miss local meaningful semantic relationships over long sequences, and the proposed model is better able to extract and preserve these relationships. For MR and SST-2, the accuracy of the proposed model was comparable to that of the other models. We propose two reasons for the weakening of the advantages of the proposed model. First, most of the samples in the two datasets are short texts, which are less dependent on the ability to extract multi-scale features than document-level texts. Second, the small sample sizes of the two datasets limit the training of the proposed model.

### 4.5. Study of PG-Res2Net

#### 4.5.1. Tuning of Hyperparameters

The position-wise gating mechanism in the PG-Res2Net module is critical to determining the performance of the proposed model. To verify the effectiveness of the gating mechanism, we conducted a comparison of the proposed model with Res2Net and the proposed model with PG-Res2Net. The comparison results are given in Table 3. The highest accuracy on each dataset was achieved by the proposed model with PG-Res2Net. Except *S* = 3 on Yelp.F and *S* = 3 on T&G, the accuracy with PG-Res2Net was higher than that with Res2Net under the same *S*. It means that the gating mechanism can select the optimized scales of features that are more effective to improve the performance of the proposed model.

Table 3 also shows how the performance is influenced by the number *S* of convolution ways of a residual block. *S* is varied among {3, 4, 5, 6}. For different datasets, the value of *S* for which the model with Res2Net accomplished the best accuracy was not fixed for different datasets, and the value of *S* for which the model with PG-Res2Net achieved the best accuracy was fixed at 4. Without the help of the gating mechanism, the selection of feature scales is more dependent on the variation of *S*. A smaller value of *S* limits the range of feature scales. While a larger value of *S* allows learning with a wider range of features, it also introduces more noise. Thus, the gating mechanism reduces the dependence of the proposed model on *S*.

#### 4.5.2. Visualization of Multi-Scale Sentiment Features

**Figure 5:**
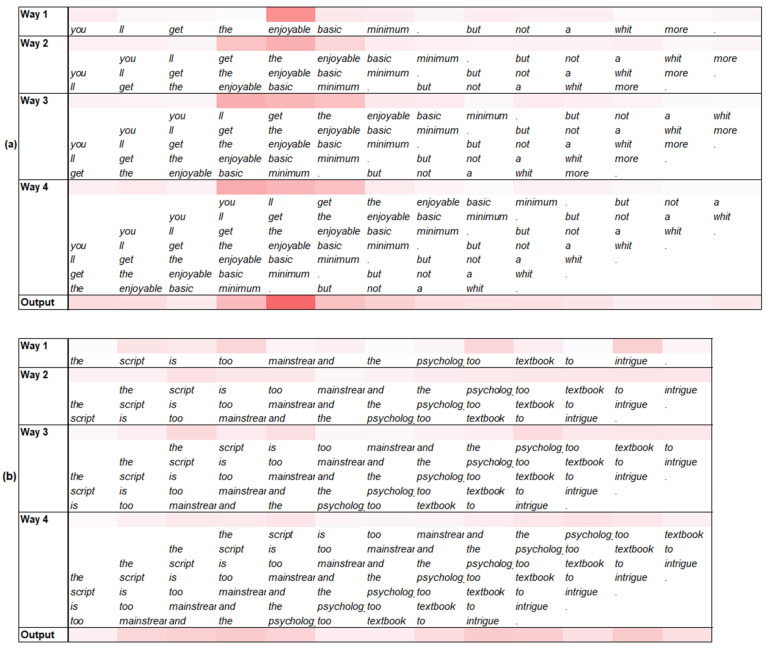
Heatmaps of multi-scale sentiment features and representations of residual block 1 in the PG-Res2Net. (**a**,**b**) show two texts with positive and negative sentiment polarities, respectively.

In this subsection, we demonstrate the effectiveness of the residual blocks of the PG-Res2Net module in the proposed model. Considering residual block 1 trained by MR as an example, Figure 5 shows the heatmaps of its multi-scale sentiment features and sentiment representations generated by the two texts. For each image, the first four rows correspond to the sentiment features extracted by the four convolution ways of the block, respectively. The upper part of each row shows the heatmap of a sentiment feature, and the lower part shows the phrases corresponding to the positions of the feature. The last row shows the heatmap of a sentiment representation. These sentiment features and representations were first transformed into intensity vectors and then visualized.

For the text shown in Figure 5a, Way 1 in the block captured “*enjoyable*” (1-scale), which has a strong positive sentiment intensity and is an important influence on the sentiment polarity of the text. Ways 2, 3, and 4 also captured the phrases of 3-scale, 5-scale, and 7-scale with strong sentiment intensity. All of these ways contain “*enjoyable*”. When the phrases including “*enjoyable*” contain the conjunction word “but” or the negative word “*not*”, their sentiment intensity is evidently weakened, such as “*enjoyable basic minimum. but*” (5-scale) and “*enjoyable basic minimum. but not a*” (7-scale). This indicates that a single residual block in the PG-Res2Net module can accurately extract sentiment features at different scales using multiple convolution ways, residual-like connections, and gates between ways.

For the text shown in Figure 5b, the sentiment intensity of each word (1-scale), which was captured by Way 1, was not very strong. Although Ways 2, 3, and 4 gradually captured more phrases with a certain sentiment intensity, such as “*the script is too mainstream*” (5-scale) and “*the psychology too textbook to intrigue*” (7-scale), their sentiment intensity is still weak. This phenomenon is not conducive to judging text sentiment polarity. However, the overall sentiment intensity of its sentiment representation is significantly enhanced and can determine the sentiment polarity of the text. This illustrates that a residual block in the PG-Res2Net module can effectively select multi-scale sentiment features to generate task-friendly sentiment representations. As mentioned in Section 3.4, the sentiment representation of a block selectively contains the multi-scale features extracted by the block.

### 4.6. Effectiveness of Selective Fusing Module

To investigate the effect of the selective fusing module in the proposed model, ablation experiments were conducted on MR and Yelp.F, which represent 2-category short-text-level and 5-category document-level datasets, respectively. The four structures were constructed as follows, and the results are listed in Table 4.

- 3-Blocks-W-SF: The structure has a PG-Res2Net module containing 3 residual blocks and a selective fusing module for MR.
- 3-Blocks-WO-SF: The structure is similar to 3-Blocks-W-SF except that an average method replaces the selective fusing module.
- 7-Blocks-W-SF: The structure has a PG-Res2Net module containing 7 residual blocks and a selective fusing module for Yelp.F.
- 7-Blocks-WO-SF: The structure is similar to 7-Blocks-W-SF except that an average method replaces the selective fusing module.

**Table 4:** Ablation study on the selective fusing module of the proposed model. Test accuracy (%) is used as an evaluation metric.

	**3-Blocks-W-SF**	**3-Blocks-WO-SF**	**7-Blocks-W-SF**	**7-Blocks-WO-SF**
MR	81.7	81.4	-	-
Yelp.F	-	-	66.5	64.3

**Figure 6:**
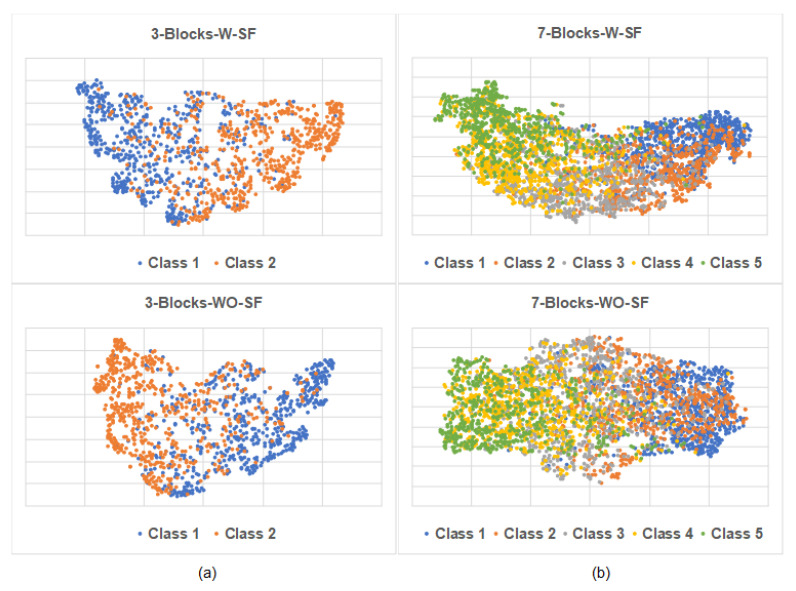
Two-dimensional t-SNE visualization of text sentiment representations. (**a**) Text sentiment representations produced by 3-Blocks-W-SF and 3-Blocks-WO-SF on MR. (**b**) Text sentiment representations produced by 7-Blocks-W-SF and 7-Blocks-WO-SF on Yelp.F.

As shown in Table 4, for both MR and Yelp.F, the removal of the selective fusing module led to a decline in accuracy, particularly for Yelp.F. We further used t-SNE to visualize the text sentiment representations of the four structures, which were the outputs of the selective fusing modules or the alternative average methods. The corresponding results are shown in Figure 6, where every point represents a sample, and different colors represent different classes. For MR, Figure 6a shows that the text sentiment representations of 3-Blocks-W-SF and 3-Blocks-WO-SF form different clusters. However, the boundary between the different clusters of 3-Blocks-W-SF is more evident than that of 3-Blocks-WO-SF. For Yelp.F, Figure 6b shows that the text sentiment representations of 7-Blocks-W-SF and 7-Blocks-WO-SF do not form different clusters well. We suggest that this phenomenon might be caused by the difficulty of multi-category document-level datasets and the similar sentiment representation projections of texts adjacent to the sentiment polarity. The clusters of Classes 2, 3, and 4 of 7-Blocks-WO-SF almost overlapped. However, the clusters of 7-Blocks-W-SF can be distinguished and distributed in space in the order of sentiment polarity. Overall, the selective fusing module can optimally select sentiment features from different levels of sentiment representations to generate a task-friendly text sentiment representation.

### 4.7. Analysis of Model Scalability

**Figure 7:**
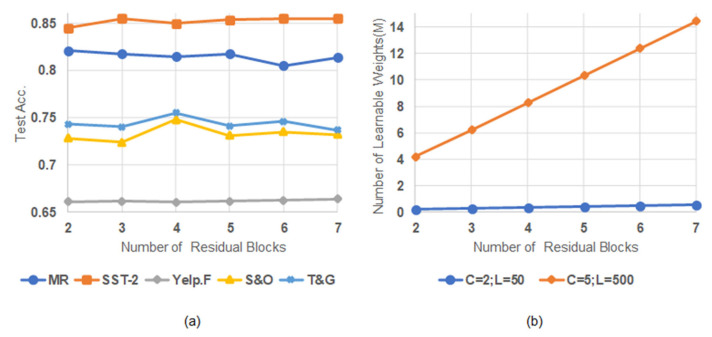
Impact with a different number of residual blocks. (**a**) Test accuracy with a different number of residual blocks. (**b**) Relationship between the number of residual blocks and the number of learnable weights.

The proposed model can better handle target datasets with different text length distributions and sample sizes by scaling the number *D* of its residual blocks. In this subsection, we assess how the scaling of the model influences its performance. The model had two forms in this experiment. When processing 2-category short-text-level datasets, *C* = 2 and *L* = 50, and when processing 5-category document-level datasets, *C* = 5 and *L* = 500. Figure 7a shows the accuracy of the model for the five datasets for different *D* values. For Yelp.F, S&O, and T&G, the accuracy continuously improved with an increase in *D* until *D* = 7, 4, and 4, respectively. This is because the three datasets are document-level and dependent on multi-scale sentiment features in a larger range, whose extraction requires more residual blocks. For the short-text-level datasets MR and SST-2, the accuracy reached the maximum when *D* = 2 and 3, respectively. We suggest that the sentiment classification of short texts depends more on small-scale sentiment features, which may be obtained using only a few residual blocks. Moreover, a single residual block in the PG-Res2Net module can learn a certain range of sentiment features. 

Figure 7a also shows that, for all datasets except Yelp.F, the accuracy begins to decrease and fluctuate when *D* exceeds a certain value. This may be caused by overfitting, which is triggered by the relatively small sample size of a training set and the more learnable weights of a model. As shown in Figure 7b, although the number of the learnable weights of the model (*C* = 5 and *L* = 50) did not increase significantly with an increase in *D*, the training sample size of MR and SST-2 were small enough to easily cause overfitting. For S&O and T&G, the training sample size satisfied the increase in the number of the learnable weights (*C* = 5 and *L* = 500) when *D* was not too large. For Yelp.F, the accuracy always increased when *D* increased from 2 to 7 because the dataset had sufficient training samples to train more learnable weights. Overall, increasing *D* within a certain range may improve the accuracy of the model for document-level datasets.

### 4.8. Error Analysis

**Figure 8:**
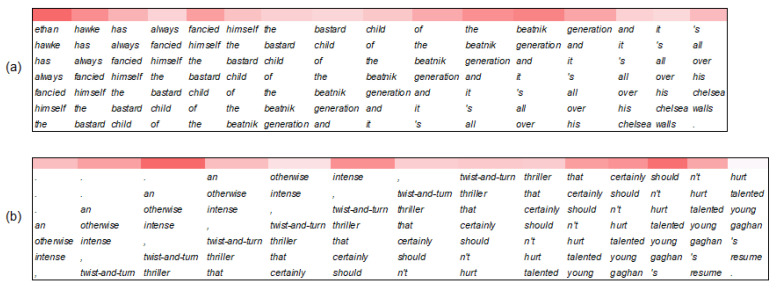
Heatmaps of representations of residual block *1* in the PG-Res2Net module. (**a**,**b**) are the representative texts of two factors that cause incorrect predictions.

An error analysis of the proposed model was conducted, and it was found that most of the errors could be summarized as follows. The first factor is a lack of background knowledge. An example is “*ethan hawke has always fancied himself the bastard child of the beatnik generation and it’s all over his chelsea walls*.”, whose representation of residual block *1* is shown in Figure 8a. As observed in the representation, the most emphasized phrase is “*ethan hawke has always fancied himself the*”. However, it does not have a strong sentiment. “*beatnik*” and “*chelsea walls*”, which are decisive for the sentiment judgment of the text, require relevant background knowledge to be understood. The second factor is the mutual interference between different sentiment tendencies in a text with less prominent sentiment, such as “*an otherwise intense, twist-and-turn thriller that certainly shouldn’t hurt talented young gaghan’s resume*.”. From Figure 8b, while the phrase “*an otherwise intense, twist-and-turn thriller*” with negative sentiment is emphasized, the phrase “*shouldn’t hurt talented young gaghan’s*” with positive sentiment is also emphasized. These two phrases with different sentiment tendencies make it difficult to judge the less prominent sentiment of the whole text.

## 5. Conclusions

In this study, a novel CNN model is proposed for sentiment analysis of short texts and documents, in which a PG-Res2Net module and a selective fusing module are defined. This model is intuitively designed to earn credible text sentiment representations through the interaction and fusion of various scale features for predicting the right sentiment of a text, where multi-scale sentiment features are achieved by developing the optimized interaction among various small-scale sentiment features. Furthermore, text sentiment representations are produced by selectively fusing multi-scale features over a large range. Compared with other CNN-based models, the proposed model can obtain more abundant multi-scale sentiment features and alleviate the loss of local detailed information caused by a convolution operation. The model achieved comparable or better performance on the five benchmark datasets compared with the other models. The comparison results, ablation studies, and visualizations also demonstrated the proposed model’s ability to optimize the interaction among multi-scale features and selectively fuse multi-scale features. 

Although this model achieves marginal improvement over other models, several research areas warrant further investigation. First, sentiment datasets often show category imbalances, and we attempt to handle the imbalances using the reuse of multi-scale sentiment features across samples. Second, there is interference between the features with different sentiment tendencies in a text with less prominent sentiment, and we try to use computational intelligence algorithms, such as monarch butterfly optimization and differential evolution, to further optimize and improve the feature selection operator. Third, there is other information associated with texts, such as user and product information [54,55], and we are exploring further how this information can be used.

## Figures and Tables

**Table 1:** Data statistics. Training, training set size; Testing, test set size; Classes, number of classes; Avg-Len, average text length; Max-Len, maximum text length.

**Dataset**	**MR**	**SST-2**	**Yelp.F**	**S&O**	**T&G**
Training	7.1 K	6.9 K	650 K	294.0 K	165.4 K
Testing	3.6 K	1.8 K	50 K	1 K	1 K
Classes	2	2	5	5	5
Avg-Len	21	19	155	99	114
Max-Len	62	56	1214	6467	6224

**Table 2:** Test accuracy (%) of the proposed model and other models on the five datasets. The results marked with * are obtained by our re-implementation.

Model		MR	SST-2	Yelp.F	S&O	T&G
RNN	Bi-LSTM [43]	79. 7	83. 2	54.8	71.9	70.7
	HAN [44]	77.1	-	-	72.3	69.1
	CAHAN [47]	-	79.8	-	73.0	70.8
	HUSN [46]	81.5 *	82.2	-	-	-
CNN	Classical CNN [11]	81.5	87.2	65.5	72.0	70.5
	AGCNN [31]	81.9	87.4	62.4	-	-
	TextConvoNet [21]	-	-	63.1	71.3 *	73.2 *
ResNet	VDCNN (29 layers) [24]	72.8	78.2	64.7	72.3 *	74.8 *
	Word-DenseNet [45]	79.6 *	82.2 *	64.5	67.6 *	72.6 *
	DenseNet with Multi-scale Feature Attention [6]	81.5	84.3 *	66.0	71.6 *	74.2 *
Transformer	SAHSSC [48]	-	-	-	73.6	72.5
	Sentiment-Aware Transformer [49]	79.5	84.3	-	-	-
This work	CNN with PG-Res2Net and Selective fusing	82.3 (*D* = 2)	85.5 (*D* = 2)	66.5 (*D* = 7)	74.8 (*D* = 4)	75.5 (*D* = 4)

**Table 3:** Test accuracy (%) of the proposed model with Res2Net and PG-Res2Net on the five datasets.

	*S*	MR (*D* = 2)	SST-2 (*D* = 2)	Yelp.F (*D* = 7)	S&O (*D* = 4)	T&G (*D* = 4)
with ResNet	3	81.0	84.4	66.1	73.3	75.0
	4	82.0	84.6	65.9	73.6	74.7
	5	81.2	83.9	66.1	72.3	74.4
	6	81.2	84.1	66.2	72.6	74.6
with PG-Res2Net	3	81.5	84.6	65.6	73.8	74.2
	4	82.3	85.5	66.5	74.8	75.5
	5	81.7	84.0	66.3	72.8	75.2
	6	82.1	84.2	66.3	73.0	74.8

## Data Availability

Not applicable.
